# F211. FINDING AND FIXING ATTENTIONAL DYSFUNCTION IN SCHIZOPHRENIA

**DOI:** 10.1093/schbul/sby017.742

**Published:** 2018-04-01

**Authors:** Robert Reinhart

**Affiliations:** Boston University

## Abstract

**Background:**

Schizophrenia is the most debilitating health problem that exists, and its cognitive impairments are the greatest predictor of disability. Since the earliest clinical descriptions of the illness, abnormalities of attention have been at the core of the cognitive symptoms of schizophrenia. Theories of attentional dysfunction in schizophrenia propose that the deficits arise from either an inability to maintain working memory representations that guide attention, or difficulty focusing lower-level visual attention mechanisms. However, these theoretical accounts neglect the role of long-term memory representations in controlling attention.

**Methods:**

To test competing accounts of the etiology of cognitive deficits in schizophrenia, we devised a cued visual search task that allowed us to examine the integrity of the memory mechanisms that control attention and the lower-level mechanisms for focusing attention on visual inputs in patients with schizophrenia and demographically matched controls. In this task, a target object was cued at the beginning of each trial. The task-relevant cue signaled the identity of the target that could appear in the search array presented a second later. Then the target remained the same for three to seven consecutive trials (length of run randomized) before it was changed to a different object. While patients and controls were repeatedly searching for the same target object, we used electrophysiological measurements of brain activity to directly measure how they were focusing attention on the search targets, as well as how they recruited working memory and long-term memory representations to control attention as they searched for the task-relevant targets. In a second experiment, we used a causal manipulation of brain activity to provide converging evidence for our hypotheses regarding the locus of the attentional deficits in schizophrenia and to determine whether it is possible to improve attention in patients. This experiment was a double-blind, sham-controlled, within-subjects design using transcranial direct-current stimulation (tDCS) and established electroencephalographic (EEG) signatures of working memory, long-term memory, and attention.

**Results:**

Here, we show that the control of perceptual attention is impaired in people with schizophrenia, and that this impairment is driven by an inability to shift attentional control from working memory to long-term memory across practice. Contrary to predictions of the dominant models, this attentional impairment is observed in the face of exuberant neural activity indexing working memory and completely normal activity indexing the focusing of visual attention. Next, we provide converging evidence for the locus of attentional impairments in long-term memory by showing that noninvasive electrical stimulation of medial frontal cortex rectifies long-term memory related neural signatures and normalizes the ability of patients to find targets in complex visual scenes.

**Discussion:**

The findings challenge existing views of the locus of dysfunction underlying attentional impairments in schizophrenia. Moreover, the results highlight the crucial importance of long-term memory systems in controlling attention and associated abnormalities in the hippocampus and other brain areas in schizophrenia.

